# Effect of aminomix-1 solution as an in vitro scolicidal agent on the bile ducts and liver in rats: a controlled experimental animal study

**DOI:** 10.1590/1516-3180.2025.3467.13052026

**Published:** 2026-08-21

**Authors:** Recep Çağlar, Hüseyin Pülat, Emine Tekingündüz, Tiinçe Aksak

**Affiliations:** I Physician; Associate Professor, Department of General Surgery, Gastroenterological Surgery, Mersin City Training and Research Hospital, Mersin, Türkiye.; II Physician; Department of General Surgery, Mersin City Training and Research Hospital Mersin, Mersin, Türkiye.; III Physician; Department of Pathology, Mersin City Training and Research Hospital Mersin, Mersin, Türkiye.; IV Assistant Professor, University of Toros Medical Services and Techniques Head of Department, Mersin, Türkiye.

**Keywords:** Parenteral Nutrition Solutions, Anticestodal Agents, Hydatid Cyst, Bile ducts, Cystobiliary Fistula, Echinococcosis

## Abstract

**BACKGROUND:**

Many scolicidal agents are used to treat hydatid cysts. However, most of these have been discontinued because of complications. Currently, no safe, effective, and useful scolicidal agent are available for the treatment of hydatid cysts in humans. Therefore, novel agents with greater efficacy, fewer side effects, and lower costs are required. In this study, we evaluated the effects of aminomix-1 solution on the liver and bile ducts of rats.

**MATERIALS AND METHODS:**

Twenty-two male Wistar-Albino rats were divided into two groups. A 0.5 mL solution of aminomix-1 was injected into the common bile duct with a 27-gauge syringe without pressure in the experimental group and normal saline (0.9% isotonic) in the control group. Blood samples for hepatic function tests and liver and bile duct tissues were examined histopathologically 4 weeks after the procedure. All statistical analyses were performed using IBM SPSS Statistics for Windows, version 21.0. Statistical significance was set at p < 0.05.

**RESULTS:**

The aminomix-1 group showed significantly higher aspartate aminotransferase and alkaline phosphatase levels than the control group. No significant differences were observed in the alanine aminotransferase and amylase levels. Total and direct bilirubin and gamma-glutamyl transferase levels were below the detection limits. No fibrosis or confluent necrosis was observed in either group. The aminomix-1 group showed moderate necrosis in the spotty (focal) necrosis analysis, which was borderline significant (p ≈ 0.08).

**CONCLUSION:**

Although aminomix-1 caused mild biochemical and histopathological changes, it did not cause advanced or irreversible liver damage or sclerosing cholangitis.

## INTRODUCTION

 Despite recent advances, hydatid cyst disease remains an important public health issue globally.^1^ The World Health Organization has called for new, effective scolicidal agents with fewer complications.^2^ Surgery remains the basis of the treatment for the hydatid cyst disease.^3^ Many chemical scolicidal agents have been used to eliminate cyst scoleces; however, most cause unwanted complications that limit their use, such as sclerosis and chemical cholangitis.^4^ Thus, surgeons urgently require novel scolicidal agents with fewer side effects, lower cost, and higher efficacy.^5^ We previously demonstrated that aminomix-1 solution is an effective scolicidal agent in vitro. In this study, we investigated the effects of aminomix-1 solution on the liver and bile ducts of rats. 

## MATERIALS AND METHODS

### Aminomix-1 formula

 Malic acid 1 (4.598 g), threonine 2 (4.4 g), L-methionine 2 (4.3 g), L-histidine 2 (3 g), L-lysine mono hydrochloride 1 (6.188 g), L-proline 1 (11.25 g), L-alanine 1 (11.25 g), L-methionine 3 (2.15 g), L-phenylalanine 3 (2.55 g), L-threonine 3 (2.20 g), L-tryptophane 3 (2.2 g), L-valine 3 (3.1 g), arginine 3 (6 g), L-valine 2 (6.2 g), L-isoleucine 3 (2.25 g), L-isoleucine 1 (3.75 g), L- leucine 3 (3.7 g), glycine 3 (7 g), L-phenylalanine 2 (5.1 g), L-lysine mono hydrochloride 3 (4.125 g), L-leucine 1 (5.55 g), L-histidine 1 (2.25 g), L-methionine 1 (3.23 g), L-phenylalanine 1 (3.83 g), L-threonine 1 (3.3 g), L-tryptophan (1.5 g), L-valine 1 (4.65 g), L-lysine mono hydrochloride 2 (8.25 g), glycine 1 (10.5 g), arginine 2 (12 g), L-histidine 3 (1.5 g), malic acid 3 (3.065 g), L-proline 2 (15 g), L-alanine 2 (15 g), malic acid 2 (6.13 g), L-leucine 2 (7.4 g), L-isoleucine 2 (5 g), glycine 2 (14 g), arginine 1 (9 g), L-tryptophan 2 (2 g), L-alanine 3 (7.5 g), L-proline 3 (7.5 g); electrolytes (mmol/L): sodium (Na+), 35.5; potassium (K+), 25; magnesium (Mg++), 2.5; chloride (Cl−), 53.4; acetate (CH_3_COO−), 25; serum osmolarity, 718 mOsmol/L. Aminomix-1 was used for total parenteral nutrition and electrolyte and fluid replacement at 20 mL/kg/day. 

 Twenty-two Wistar-Albino male rats (200 ± 15 g) were used. The rats were fasted for 12 h before anesthesia but had free access to water until 2 h before anesthesia. Preoperative enteral antibiotics and analgesics were administered. Animals were housed individually at 21^°^C ± 2 ^°^C with a 12-h light–dark cycle. Two rats that died postoperatively in the experimental and control groups were excluded from the study. Upon review, the deaths were due to perioperative complications rather than the administered solution; however, a definitive cause was not established. Owing to the exploratory nature, no a priori power analysis was performed. This study was approved by the Ethics Committee on the Care of Animals at the Mersin University Faculty of Medicine Experimental Animals Research Laboratory (Ethics Committee No. 2024/23). 

### Surgical procedure

 The rats were randomly divided into two groups. Anesthesia was induced using intramuscular ketamine hydrochloride (40 mg/kg) and xylazine (5 mg/kg). All animals were allowed to breathe spontaneously. 

 After shaving and cleaning the abdomen with povidone-iodine, midline laparotomy was performed. In the experimental group, 0.5 mL of aminomix-1 solution was injected into the common bile duct using a 27-gauge syringe without pressure. The control group received normal saline (0.9% isotonic). Immediately after the injection, the common bile duct was clamped for 3 min using a non-traumatic vascular clamp (bulldog) and then allowed to drain. 

 A second midline laparotomy was performed under ketamine hydrochloride anesthesia 4 weeks postoperatively. Blood samples were drawn to assess the levels of bilirubin, aspartate aminotransferase (AST), alanine aminotransferase (ALT), alkaline phosphatase (ALP), gamma-glutamyl transferase (GGT), and amylase. The liver and bile ducts were excised en bloc, fixed in 10% formalin, and embedded in paraffin. The sections were stained with hematoxylin and eosin and examined under a light microscope for histopathological changes. Hepatic and bile duct injuries were assessed histomorphologically. Confluent necrosis, interface hepatitis, portal inflammation, bile duct injury, fibrosis, and necrosis were graded independently. Histopathological evaluations were performed by a pathologist who was blinded to the group allocation. An autoanalyzer (Siemens ADVIA) was used for biochemical tests. 

### Statistical Analysis

 All statistical analyses were performed using IBM SPSS Statistics for Windows version 21.0 (IBM Corp., Armonk, NY, USA). The distribution of numerical variables was evaluated using the Shapiro–Wilk test. The results are reported as mean ± standard deviation (mean ± SD) for normally distributed data and median (minimum–maximum) for non-normally distributed data. An independent-samples Mann–Whitney U test was used to compare the distribution characteristics between the two groups. 

 The Mann–Whitney U test was used for the ordinal data. Categorical variables are expressed as numbers (percentages) and were compared using Fisher’s exact test between the two groups. Statistical significance was set at p < 0.05. 

## RESULTS

### Biochemical results

 As not all parameters met the criteria for normal distribution (Shapiro–Wilk test), the groups were compared using the Mann–Whitney U test for biochemical variables. The Welch test results were provided for parameters with near-normal distributions (ALP). The threshold for statistical significance was set at p < 0.05. The effect size for the parametric distributions was calculated using Cohen’s d coefficient. 

 AST and ALP levels were significantly higher in the aminomix-1 group than in the control group (Mann–Whitney p = 0.0046 and 0.0022, respectively). No significant differences were observed in ALT and amylase levels. The effect size for ALP was high (Cohen’s d ≈ 1.85), indicating a significant difference between the groups. As the total and direct bilirubin levels and GGT levels were below the detection threshold in all rats (< 0.1 mg/dL and < 6 U/L), no statistical comparison was performed (**Table 1**). 

**Table 1:** Biochemical findings and statistical test results

**Parameter**	**Group (n = 10)**	**Median [IQR]**	**Mean ± SD**	**Mann–Whitney p**	**Welch test p**	**Effect size (Cohen’s d)**
AST (U/L)	Aminomix	155 [55.75]	182.6 ± 66.1	0.0046	0.0716	0.86
	Control	110 [15.75]	129.9 ± 56.4			
ALT (U/L)	Aminomix	84 [81.5]	105.1 ± 51.7	0.3443	0.155	0.68
	Control	73.5 [20.25]	78.6 ± 18.9			
ALP (U/L)	Aminomix	149 [57.25]	163.8 ± 37.5	0.0022	0.0021	1.85
	Control	112.5 [10.25]	113.5 ± 8.7			
Amylase (U/L)	Aminomix	1451.5 [107.25]	1437.9 ± 75.9	0.8501	0.7717	−0.13
	Control	1420.5 [98.25]	1452.1 ± 131			

### Histopathological results

 Pathological findings were scored semi quantitatively as absent (0), mild (+), moderate (++), or severe (+++). Nonparametric tests are recommended for small samples and nominal or ordinal data. Fisher’s exact test was used to compare categorical variables in two independent groups when the expected frequencies of the cells were < 5. 

**Figure 1:**
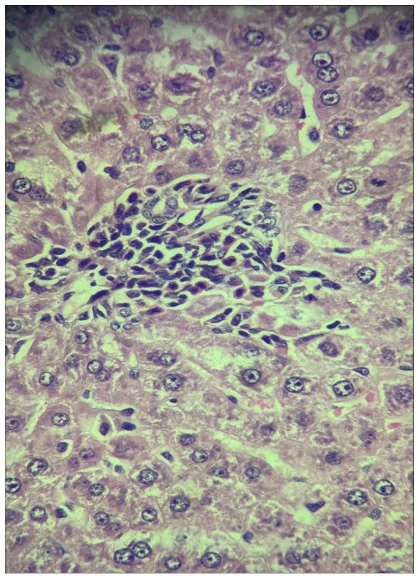
Interface

**Figure 2 F2:**
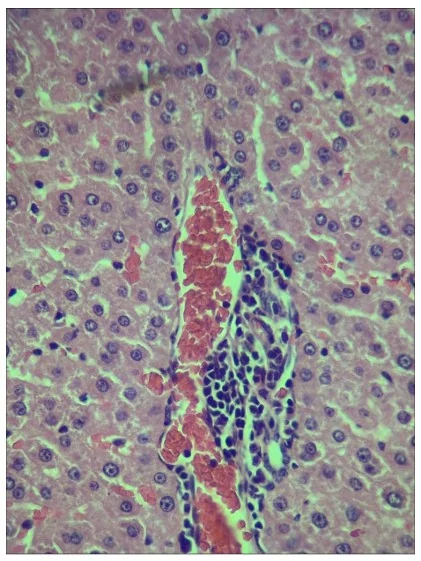
Portal inflammation

**Figure 3 F3:**
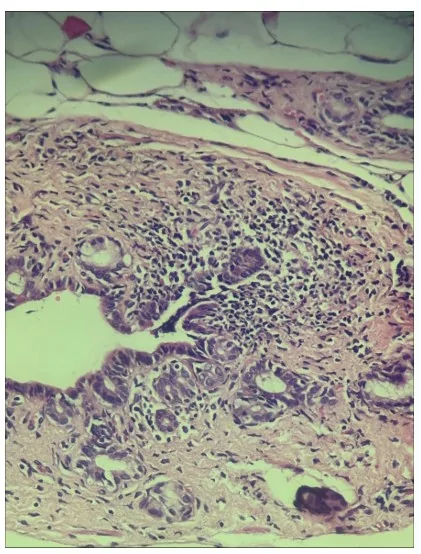
Main bile duct inflammation

**Figure 4 F4:**
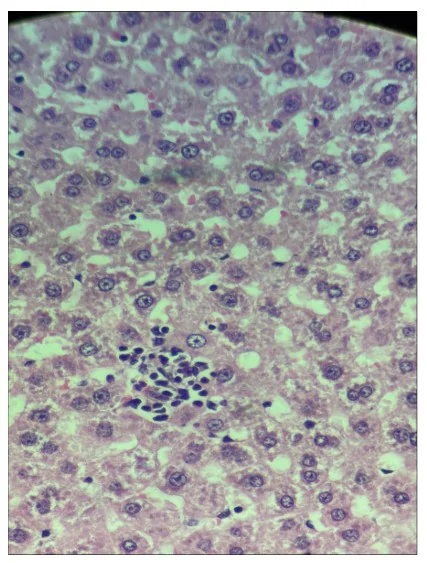
Spotty necrosis

 Fibrosis and confluent necrosis were absent in both groups (all values were 0); therefore, no comparison was performed. Inflammation and bile duct interface findings were analyzed in a binary manner as “present/absent” (0 or +). Portal inflammation and spotty (focal) necrosis scores were quantified as + = 1, ++ = 2; none of the samples showed a +++ value (**Figures 1-4** [Fig F2] [Fig F3] [Fig F4]). 

 Seven rats in the aminomix-1 group showed mild spotty (focal) necrosis, and three showed moderate necrosis. This finding showed borderline significance when compared with that of the control group (Mann–Whitney U test, p = 0.08). This may reflect an aminomix-1 effect but requires verification in a larger sample (**Table 2**). 

**Table 2:** Pathological findings and statistical test results

**Pathological finding**	**Control distribution (n = 10)**	**Aminomix distribution (n = 10)**	**Test**	**p value**	**Interpretation**
Bile duct inflammation	5 no inflammation, 5 mild	3 no inflammation, 7 mild	Fisher’s exact	0.65	Not significant
Portal inflammation	10 mild	9 mild, 1 moderate	Mann–Whitney U	0.37	Not significant
Spotty (focal) necrosis	10 mild	7 mild, 3 moderate	Mann–Whitney U	0.08	Borderline; trend toward higher scores
Interface	10 absent	9 absent, 1 mild	Fisher’s exact	10	Not significant

Note: Fisher’s exact test was used for binary variables (presence/absence), and the Mann–Whitney U test was used for ordinal variables.

## DISCUSSION

 Currently, no ideal non-invasive treatment exists for hydatid cyst disease. Surgery is the first-line therapy; however, despite the advantages of surgical techniques, there is a risk of leaks and secondary infections. Current treatment methods include conventional medicine, percutaneous aspiration, and surgery.^6^ Among them, the puncture–aspiration–injection–reaspiration (PAIR) technique as a percutaneous aspiration treatment has drawn interest as a minimally invasive technique, particularly for the treatment of Gharbi type 1,2 hydatid cysts.^7^ Thus, checking for parasitic seedlings during surgery is important. Many surgeons use scolicidal agents to prevent parasitic seedlings during surgery. However, leakage of cyst fluid containing scoleces during surgery may cause disease recurrence at a rate of 3–30%.^8^

 Savlon, hypertonic saline, silver nitrate, chlorhexidine, formaldehyde, ethyl alcohol, hydrogen peroxide, *Eucalyptus globulus, Mesobuthus eupeus toxin*, and betadine are used as scolicidal agents worldwide, though many were discontinued due to early- and late-term complications.^9 -12^ Although the WHOIWGE (Informal Working Group on Echinococcosis) recommends the use of 20% hypertonic saline as a scolicidal agent during surgery or 95% ethyl alcohol during PAIR, comparative studies are required.^13,14^

 Sclerosing cholangitis (SC) is the most common and feared complication following cyst leakage and dissemination of various scolicidal agents. Immunological reactions drive the pathogenesis of SC. Direct bile duct exposure to scolicidal agents is a primary cause of secondary SC.^15,16^ Several studies have revealed the scolicidal effects of Ag-nanoparticles synthesized from the liquid volatile extract of Penicillium aculeatum, methanolic extract of Sambucus ebulus fruit, nitric oxide metabolites, and endophytic Pestalotiopsis against scoleces.^17-20^ Malekifard et al.^21^ reported that gold nanoparticles at a concentration of 1 mg/mL killed all scoleces within 60 min. In our previous in vitro study, we showed that silver nitrate, dextrose, isotonic saline, and mannitol demonstrated 100% scolicidal activity after 20, 30, 45, and 45 min, respectively.^7^ Mahmoudvand et al.^22^ showed that biogenic selenium nanoparticles were effective at 500 and 250 μg/mL within 10 and 20 min, respectively. Eskandarian et al.,^23^ demonstrated that the chloroform extract of garlic could kill 98% of the scoleces at 50 mg/mL and after a 20 min. 

 Most hydatid cysts are connected to the bile ducts, known as cystobiliary fistulas. Thus, it is important that scolicidal agents do not cause SC. If a cystobiliary fistula and cyst fluid is bile-colored, hypertonic saline and silver nitrate should be avoided.^17,18^ Hosseini et al. showed that 5% hypertonic saline and 1% eucalyptus essential oil caused intra- and extrahepatic bile duct injury and portal inflammation, potentially causing long term SC.^24^ Houry et al.^25^ showed that 5% hypertonic saline solution had minimal or no effect on SC induction. 

 Hypertonic saline solution, a common scolicidal agents, may cause hypernatremia, as diagnosed in studies using 20% and 30% solutions.^26,27^ Ethyl alcohol may cause SC in patients with cystobiliary fistula, and this effect is concentration-dependent.^28^ Betadine and povidone-iodide may cause complications, such as depot disease, kidney failure, sterile peritonitis, and sclerosing serositis.^29^ Cetrimide causes complications, such as sclerosing peritonitis, metabolic acidosis, and methemoglobinemia.^30,31^

 In our study, AST and ALP levels were significantly higher in the aminomix-1 group than in the control group (Mann–Whitney U test, p = 0.0046 and 0.0022, respectively). No significant differences were observed in ALT, amylase, total and direct bilirubin, and GGT levels. 

 Histopathologically, no fibrosis or confluent necrosis was observed in the aminomix-1 and control groups. No significant differences were observed between the groups in terms of bile duct, interface, or portal inflammation. 

 Mild spotty (focal) necrosis occurred in seven rats and moderate spotty (focal) necrosis in three rats in the aminomix-1 group. This finding showed borderline significance compared to the control group (Mann–Whitney U test, p ≈ 0.08), potentially indicating a necrotic effect of aminomix-1; however, this should be confirmed using a larger sample. 

 Aminomix-1 is a hyperosmolar solution used for total parenteral nutrition. Hyperosmolar formulations exert osmotic stress on the biliary epithelium, potentially leading to cellular irritation, altered bile composition, and impaired bile flow. These effects may contribute to cholestatic changes and elevated cholestatic enzymes, such as ALP.^32^

 In this study, the significantly higher ALP levels in the aminomix-1 group and more frequent mild bile duct inflammation may be partially explained by the osmolar characteristics of the administered solution. 

## CONCLUSION

 To date, no safe, effective, or useful scolicidal agent has been developed for hydatid cysts in humans. As many scolicidal agents cause SC in bile ducts, their use is limited. In conclusion, aminomix-1 administration in this experimental model did not result in advanced fibrosis or severe bile duct injury. However, elevated liver enzymes and mild-to-moderate hepatocellular necrosis suggest hepatic stress. These findings indicate that while aminomix-1 may be relatively well tolerated, its safety profile requires investigation in studies with larger sample sizes and longer follow-up periods. 

### Limitations of the Experimental Model

1. The relatively small sample size may have limited statistical power, increasing the risk of Type II errors, particularly for histopathological outcomes. This may explain the borderline significance of spotty necrosis (p ≈ 0.08).
2. The experimental model (direct bile duct injection with temporary clamping) does not fully replicate the physiological or clinical conditions. This approach may introduce non-physiological pressure changes and localized exposure effects that differ from those of systemic administration. Future studies using models that closely mimic clinical administration routes are required to define the hepatobiliary effects of these formulations.
3. Inability to adequately assess cholestatic injury: total and direct bilirubin and GGT levels were below the detection threshold in all rats.
4. Two animals died postoperatively and were excluded. After review, these deaths were likely related to perioperative complications rather than the administered solution; however, a definitive cause could not be established.

## Data Availability

The data supporting the findings of this study are available upon reasonable request from the corresponding author, Recep Çağlar.

## References

[1] Pawłowski ZS, Eckert J, Vuitton DA, Eckert J, Gemmell MA, Xeslin FX, Pawlowski ZS, World Health Organization (WHO), World Organisation for Animal Health (WOAH) (2001). WHO/OIE Manual on Echinococcosis in Humans and Animals: a Public Health Problem of Global Concern [Internet].

[2] Kohansal MH, Nourian A, Rahimi MT (2017). Natural products applied against hydatid cyst protoscolices: A review of past to present. Acta Tropica.

[3] Li H, Shao Y, Aji T (2014). Laparoscopic approach for total cystectomy in treating hepatic cystic echinococcosis. Parasite.

[4] Kozak O, Gulec B, Uzar A (2000). Effects of scolocidal substances on the liver and biliary tract. Gulhane Medical Journal.

[5] Adas G, Arikan S, Kemik O (2009). Use of albendazole sulfoxide, albendazole sulfone, and combined solutions as scolicidal agents on hydatid cysts (in vitro study). World J Gastroenterol.

[6] Moazeni M, Larki S (2010). In vitro effectiveness of acidic and alkaline solutions on scolices of hydatid cyst. Parasitol Res.

[7] Caglar R, Yuzbasioglu MF, Bulbuloglu E (2008). In vitro effectiveness of different chemical agents on scolices of hydatid cyst. J Invest Surg.

[8] Goja S, Saha SK, Yadav SK, Tiwari A, Soin AS (2018). Surgical approaches to hepatic hydatidosis ranging from partial cystectomy to liver transplantation. Ann Hepatobiliary Pancreat Surg.

[9] Mansourian S, Sadjjadi SM, Hosseini SV (2009). Evaluation of different chemical agents on the germinative layer of sheep hydatid cyst after implantation to peritoneal cavity of BALB/c. J Invest Surg.

[10] Moazeni M, Hosseini SV, Al-Qanbar MH, Alavi AM, Khazraei H (2019). In vitro evaluation of the protoscolicidal effect of Eucalyptus globulus essential oil on protoscolices of hydatid cyst compared with hypertonic saline, povidone iodine and silver nitrate. Journal of Visceral Surgery.

[11] Jafari H, Nemati M, Haddad Molayan P (2019). Scolicidal activity of Mesobuthus eupeus venom against the protoscolices of Echinococcus granulosus. Arch Razi Inst.

[12] Labsi M, Soufli I, Amir ZC, Touil-Boukoffa C (2022). Hepatic inflammation and liver fibrogenesis: A potential target for the treatment of cystic echinococcosisassociated hepatic injury. Acta Trop.

[13] Fraser I, Neoptolemos J, Darby H, Bell PR (1984). The effects of intralipid and heparin on human monocyte and lymphocyte function. JPEN J Parenter Enteral Nutr.

[14] Busto Bea V, Barrio Andrés J, Almohalla Álvarez C (2016). Liver hydatid disease: Still a problem. Medicina Clínica (English Edition).

[15] Hosseini SV, Kumar PV, Bagheri MH (2005). Incidence of Sclerosing Cholangitis Induced by Silver Nitrate in Rabbit. Journal of Applied Animal Research.

[16] Rinaldi F, Brunetti E, Neumayr A (2014). Cystic echinococcosis of the liver: A primer for hepatologists. World Journal of Hepatology.

[17] Eser I, Karabag H, Gunay S (2014). Surgical approach for patients with unusually located hydatid cyst. Ann Ital Chir.

[18] Jan Z, Zeb S, Shoaib A (2019). Hydatid cyst involving right pectoralis major muscle: a case report. International Journal of Surgery Case Reports.

[19] Verma VC, Gangwar M, Yashpal M, Nath G (2013). Anticestodal activity of endophytic Pestalotiopsis sp. on protoscoleces of hydatid cyst Echinococcus granulosus. BioMed Research International.

[20] Kirshenbaum AS, Kettelhut BV, Metcalfe DD, Garriga MM (1989). Mastocytosis in infants and children: recognition of patterns of skin disease. Allergy Proc.

[21] Malekifard F (2017). Scolicidal effect of the gold nanoparticle on protoscoleces of hydatid cyst in vitro. J Urmia Univ Med Sci.

[22] Mahmoudvand H, Dezaki ES, Kheirandish F (2014). Scolicidal effects of black cumin seed (Nigella sativa) essential oil on hydatid cysts. Korean J Parasitol.

[23] Eskandarian A (2012). Scolicidal effects of squash (Corylus spp) seeds, hazel (Curcurbia spp) nut and garlic (Allium sativum) extracts on hydatid cyst protoscolices. J Res Med Sci.

[24] Hosseini SV, Al-Qanbar MH, Khazraei H (2020). Evaluation the effects of eucalyptus essential oil and hypertonic saline as scolicidal agents in induction of sclerosing cholangitis in rabbits. Adv Biomed Res.

[25] Houry S, Languille O, Huguier M (1990). Sclerosing cholangitis induced by formaldehyde solution injected into the biliary tree of rats. Arch Surg.

[26] Albi A, Baudin F, Matmar M, Archambeau D, Ozier Y (2002). Severe hypernatremia after hypertonic saline irrigation of hydatid cysts. Anesthesia & Analgesia.

[27] Krige JEJ, Millar AJW, Rode H, Knobel D (2002). Fatal hypernatraemia after hypertonic Saline irrigation of hepatic hydatid cysts. Pediatr Surg Int.

[28] Castellano G, Moreno-Sánchez D, Gutiérrez J (1994). Report of four cases and a cumulative review of the literature. Hepato-gastroenterology.

[29] LeVeen HH, LeVeen RF, LeVeen EG (1993). The mythology of povidone-iodine and the development of self-sterilizing plastics. Surg Gynecol Obstet.

[30] Momblano P, Pradere B, Jarrige N, Concina D, Bloom E (1984). Metabolic acidosis induced by cetrimonium bromide. The Lancet.

[31] Baraka A, Wakid N, Yamout F (1980). Methemoglobinemia during surgical excision of hydatid cyst. Middle East J Anaesthesiol.

[32] Diamond IR, Sterescu A, Pencharz PB, Kim JH, Wales PW (2009). Changing the paradigm: omegaven for the treatment of liver failure in pediatric short bowel syndrome. Journal of Pediatric Gastroenterology and Nutrition.

